# Molecular Mechanisms of Plant Regeneration from Differentiated Cells: Approaches from Historical Tissue Culture Systems

**DOI:** 10.1093/pcp/pcac172

**Published:** 2022-12-22

**Authors:** Hatsune Morinaka, Duncan Coleman, Keiko Sugimoto, Akira Iwase

**Affiliations:** Center for Sustainable Resource Science, RIKEN, 1-7-22 Suehiro-cho, Tsurumi-ku, Yokohama, Kanagawa, 230-0045 Japan; Center for Sustainable Resource Science, RIKEN, 1-7-22 Suehiro-cho, Tsurumi-ku, Yokohama, Kanagawa, 230-0045 Japan; Center for Sustainable Resource Science, RIKEN, 1-7-22 Suehiro-cho, Tsurumi-ku, Yokohama, Kanagawa, 230-0045 Japan; Department of Biological Sciences, Graduate School of Science, The University of Tokyo, Hongo 7-3-1, Bunkyo-ku, Tokyo, 113-0033 Japan; Center for Sustainable Resource Science, RIKEN, 1-7-22 Suehiro-cho, Tsurumi-ku, Yokohama, Kanagawa, 230-0045 Japan; Japan Science and Technology Agency (JST), Precursory Research for Embryonic Science and Technology (PRESTO), 7 Gobancho, Chiyoda-ku, Tokyo, 102-0076 Japan

**Keywords:** Callus formation, Cell reprogramming, Organ regeneration, Somatic embryogenesis, Stem cell reformation, *Torenia fournieri*

## Abstract

Plants can exert remarkable capacity for cell reprogramming even from differentiated cells. This ability allows plants to regenerate tissues/organs and even individuals in nature and *in vitro*. In recent decades, Arabidopsis research has uncovered molecular mechanisms of plant regeneration; however, our understanding of how plant cells retain both differentiated status and developmental plasticity is still obscure. In this review, we first provide a brief outlook of the representative modes of plant regeneration and key factors revealed by Arabidopsis research. We then re-examine historical tissue culture systems that enable us to investigate the molecular details of cell reprogramming in differentiated cells and discuss the different approaches, specifically highlighting our recent progress in shoot regeneration from the epidermal cell of *Torenia fournieri*.

## Introduction

The development of multicellular organisms is achieved through coordinated cell division and differentiation to derive multiple tissues composed of specialized, heterogeneous cellular populations from a single fertilized egg. This specialization is often irreversible, and cellular totipotency is gradually lost as differentiation proceeds. Some differentiated plant cells, however, undergo reprogramming to revert to less differentiated states and exert pluripotency or totipotency in response to external stimuli such as wounding and exogenous phytohormones. Formation of callus, unorganized cell mass that contains pluripotent/totipotent cells, and *de novo* organogenesis in response to wounding or pathogen infection are observed in nature (Ikeuchi et al. 2013). After Skoog and Miller reported that the callus growth and organ regeneration from tobacco explants could be controlled by adding two types of phytohormone, cytokinin and auxin, to the medium (Skoog and Miller 1957), various *in vitro* tissue culture systems have been established to seek optimized conditions for tissue regeneration. For example, the two-step culture system in which tissue explants are cultured on an auxin-rich callus induction medium (CIM) and then transferred to a cytokinin-rich shoot induction medium (SIM) has been applied to various plant species (Nishi et al. 1968, Christianson and Warnick 1983, Koornneef et al. 1987, Coleman and Ernst 1990). In the 1950s, a pioneering study of somatic embryogenesis, the regeneration of an embryo from differentiated somatic cells, was reported using tissue culture techniques (Steward et al. 1958). Whole plant regeneration is also possible from a single protoplast, a plant cell without its cell wall, in phytohormone-containing tissue culture conditions (Nagata and Takebe 1971). Both somatic embryogenesis and whole plant regeneration from protoplasts provide clear evidence to demonstrate the plant cells’ ability to exert totipotency even after differentiation.

The drastic change in cell fates from differentiated states in plants allows us to address the following two big questions: What molecular mechanisms underlie the ability to exert cellular totipotency/pluripotency from differentiated cells? How do some differentiated plant cells maintain developmental plasticity to be reprogrammed and a differentiated state simultaneously? In this mini-review, we briefly summarize key regulators of cell reprogramming in Arabidopsis (**Table 1**). We then introduce several tissue culture systems where the reprogramming of differentiated cells is efficiently induced from non-model plants, particularly by our recent Torenia tissue culture approach (**Table 2**). We also discuss how to tackle key questions by researching non-model plants to understand the mechanisms of cell reprogramming in differentiated cells.

**Table 1:** Major key factors of cell reprogramming in Arabidopsis highlighted in this review.

Common name	Locus	Tissue culture system	References
WIND1	AT1G78080	Wound-induced cell reprogramming Two-step tissue culture	Iwase et al. (2011, 2017, 2021)
ESR1	AT1G12980	Wound-induced cell reprogramming Protoplast culture	Iwase et al. (2017) and Xu et al. (2021)
ERF115	AT5G07310	Wound-induced cell reprogramming	Heyman et al. (2016)
ALF4	AT5G11030	Two-step tissue culture Protoplast culture	Shang et al. (2016), Sugimoto et al. (2010) and Chupeau et al. (2013)
SLR/IAA14	AT4G14550	Two-step tissue culture	Shang et al. (2016)
ARF7	AT5G20730	Two-step tissue culture Protoplast culture	Fan et al. (2012) and Sakamoto et al. (2022)
ARF19	AT1G19220	Two-step tissue culture Protoplast culture	Fan et al. (2012) and Sakamoto et al. (2022)
LBD16	AT2G42430	Two-step tissue culture	Fan et al. (2012)
LBD17	AT2G42440	Two-step tissue culture	Fan et al. (2012)
LBD18	AT2G45420	Two-step tissue culture	Fan et al. (2012)
LBD29	AT3G58190	Two-step tissue culture	Fan et al. (2012)
PLT1	AT3G20840	Two-step tissue culture	Kareem et al. (2015)
PLT2	AT1G51190	Two-step tissue culture	Kareem et al. (2015)
PLT3	AT5G10510	Two-step tissue culture Wound-induced cell reprogramming Protoplast culture	Kareem et al. (2015), Ikeuchi et al. (2017), Iwase et al. (2021) and Sakamoto et al. (2022)
PLT5	AT5G57390	Two-step tissue culture Wound-induced cell reprogramming Protoplast culture	Kareem et al. (2015), Ikeuchi et al. (2017), Iwase et al. (2021) and Sakamoto et al. (2022)
PLT7	AT5G65510	Two-step tissue culture Wound-induced cell reprogramming	Kareem et al. (2015), Ikeuchi et al. (2017) and Iwase et al. (2021)
WOX5	AT3G11260	Two-step tissue culture	Zhai and Xu (2021)
ATXR2	AT3G21820	Two-step tissue culture	Lee et al. (2017)
LDL3	AT4G16310	Two-step tissue culture	Ishihara et al. (2019)
HTR15	AT5G12910	Two-step tissue culture Wound-induced cell reprogramming	Yan et al. (2020)
WUS	AT2G17950	Protoplast culture	Xu et al. (2021)
MYB3R1	AT4G32730	Protoplast culture	Sakamoto et al. (2022)
MYB3R4	AT5G11510	Protoplast culture	Sakamoto et al. (2022)
YUC1	AT4G32540	Protoplast culture	Sakamoto et al. (2022)

**Table 2:** Reprogramming from differentiated cells in various plant species highlighted in this review.

Species	Original tissues	Regenerated organs	References
N. tabacum	Epidermis	A whole plant via callus	Davey et al. (1974)
N. tabacum	Mesophyll	A whole plant via callus	Avivi et al. (2004) and Williams et al. (2003)
L. corniculatus	Root hair	A whole plant via callus	Rasheed et al. (1990)
T. fournieri	Epidermis	Shoot	Chlyah (1974)
B. rex	Epidermis	Shoot	Chlyah and Van (1975)
N. lynchii	Epidermis	Shoot and root	Venverloo et al. (1983)
D. glomerata	Mesophyll	Somatic embryo	Conger et al. (1983)
Hybrid Cichorium (C. intybus L. × C. endivia L.)	Mesophyll	Somatic embryo	Dubois et al. (1991)
D. carota	Epidermis	Somatic embryo	Nishiwaki et al. (2000)
P. amabilis	Epidermis	Somatic embryo	Chen and Chang (2006) and Kuo et al. (2005)

## Key Factors in Wound-Induced Cell Reprogramming

Applying stress treatments and tissue culture techniques to the model plant, *Arabidopsis thaliana*, has uncovered molecular mechanisms underlying the plant cell reprogramming and stem cell formation, such as reacquisition of mitotic activity and competency for *de novo* organogenesis (Ikeuchi et al. 2013, 2016, 2019). Transcriptomic analysis of Arabidopsis hypocotyl segments revealed that wounding triggers global changes in gene expression, 80% of Arabidopsis genes expressed at least once within 24 h. These include genes involved in stress response, metabolic processes, protein synthesis, cell cycle, cytokinin biosynthesis, etc. (Ikeuchi et al. 2017). An APETALA2 (AP2)/ETHYLENE RESPONSE FACTOR (ERF) family transcription factor, WOUND-INDUCED DEDIFFERENTIATION 1 (WIND1) and its close homologs, WIND2–4, were identified as regulators of cell reprogramming after wounding. They are transcriptionally activated near the wound sites and trigger callus formation and regeneration by promoting cytokinin response and the expression of various developmental regulators, including *ENHANCER OF SHOOT REGENERATION 1* (*ESR1*), leading to stem cell formation (Iwase et al. 2011, 2017, 2021). ERF115, another AP2/ERF transcription factor, also regulates wound-induced cell reprogramming, and it functions in a pathway upstream of WIND1 in the context of root tip regeneration (Heyman et al. 2016).

Epigenetic studies on Arabidopsis cell reprogramming provide crucial insights into how the expression of many of these key factors is controlled during phase changes of regeneration (Lee and Seo 2018). POLYCOMB REPRESSIVE COMPLEX 2 (PRC2) catalyzes histone H3 lysine 27 trimethylation (H3K27me3) deposition, and its loss-of-function mutation causes reprogramming of differentiated somatic cells. Given that *WIND1, WIND2, WIND3* and other reprogramming-related genes are ectopically expressed in the mutant, the PRC2 complex likely prevents reprogramming by suppressing the misexpression of reprogramming regulators in normal development (Ikeuchi et al. 2015). In the context of wounding, H3K27me3 removal is thought to be achieved at the wound site by deposition of histone H3 variant, HISTONE THREE RELATED 15 (HTR15), which has amino acid substitution and lacks K27. This atypical histone variant deposition leads to misexpression of reprogramming-related genes, such as *WUSCHEL (WUS)-RELATED HOMEOBOX 11* (*WOX11*), and results in promotion of callus formation (Yan et al. 2020). Another comprehensive analysis of changes in histone modification after wounding revealed the correlation between wound-induced gene expression and changes in the status of epigenetic modification, especially H3K9/K14 acetylation (Rymen et al. 2019). Histone acetylation inhibitor treatment suppresses both histone acetylation and gene expression of reprogramming-related transcription factors, including *WIND1*, suggesting that histone acetylation promotes wound-induced transcriptional activation and cell reprogramming in Arabidopsis.

## Key Regulators in the Two-Step Tissue Culture System

The CIM-SIM two-step tissue culture system in Arabidopsis (Valvekens et al. 1988, Akama et al. 1992) has been used to identify many regulators of reprogramming and stem cell formation. One of the characteristics of this system is that cell reprogramming leading to callus formation starts mainly from the pericycle or pericycle-like cells, which retain a capacity for cell division during lateral root formation (Atta et al. 2009, Sugimoto et al. 2010). It is thus thought that this involves reprogramming from relatively undifferentiated cells. Consistently, callus formation in this system is dependent on lateral root developmental pathways since mutation in regulators of lateral root formation, such as *ABERRANT LATERAL ROOT FORMATION* 4 (*ALF4*), abolishes the competence for callus formation (Sugimoto et al. 2010, Shang et al. 2016). Several *LATERAL ROOT BOUNDARY* (*LBD*) genes, *LBD16* and *LBD18*, which code for essential transcription factors during cell division regulation of founder cells within lateral root primordia, are also involved in auxin-induced callus formation downstream of auxin signaling by AUXIN RESPONSE FACTOR 7 (ARF7), ARF19 and SOLITARY‐ROOT/INDOLE-3-ACETIC ACID INDUCIBLE 14 (SLR/IAA14) (Fan et al. 2012, Shang et al. 2016). Indeed, the *slr-1* mutation, gain-of-function of SLR that represses ARF7/ARF19 function, shows less callus formation in the root (Iwase et al. 2011, Shang et al. 2016), and the overexpression of *LBD* genes, *LBD16, LBD17, LBD18* and *LBD29*, is able to induce callus on phytohormone-free medium (Fan et al. 2012). Moreover, stem cell formation in this auxin-rich CIM condition is achieved by root meristematic genes. Root stem cell regulators, PLETHORA 1 (PLT1) and PLT2, are induced by PLT3, PLT5 and PLT7 to acquire pluripotency in this callus (Kareem et al. 2015). Interestingly, the *plt 3,5,7* triple mutant shows a size reduction of wound-induced callus in both hypocotyl and petiole (Ikeuchi et al. 2017, Iwase et al. 2021), implying that common reprogramming regulation pathways through PLTs exist in both wound-induced callus formation and pluripotent acquisition in the callus formed in the CIM condition.

Recently, single-cell transcriptome analyses were applied to the Arabidopsis two-step culture system and identified several cellular groups inside the CIM callus (Zhai and Xu 2021). This study revealed that pluripotency is acquired in a cellular group in the middle cell layer of the callus that has a quiescent center-like transcriptional profile. In this cellular group, a root stem cell regulator, WOX5, promotes auxin production with PLT1 and PLT2 and increases sensitivity to cytokinin by repressing type-A ARABIDOPSIS RESPONSE REGULATORs (ARRs) in the direct interaction with a type-B ARR, ARR12 (Zhai and Xu 2021).

Epigenetic analyses also revealed transcriptional control of reprogramming regulators in response to auxin. For example, a histone methyltransferase, ARABIDOPSIS TRITHORAX-RELATED 2 (ATXR2) that promotes the accumulation of histone H3 lysine 36 trimethylation (H3K36me3), positively affects the *LBD16* and *LBD29* expression in combination with auxin response factors, ARF7 and ARF19 (Lee et al. 2017). LYSINE-SPECIFIC DEMETHYLASE 1-LIKE 3 (LDL3) specifically demethylates histone H3 dimethylated lysine 4 (H3K4me2) and primes gene expression for shoot regeneration in the auxin-rich CIM condition (Ishihara et al. 2019). Combinational profiling of chromatin accessibility, histone modifications and transcriptomes during the two-step culture demonstrated that the high-auxin environment during callus induction increases the chromatin accessibility of genes involved in stem cell formation (Wu et al. 2022). In this study, analysis of *arr1, arr10* and *arr12* mutants further demonstrated the role of cytokinin signaling in stem cell formation.

It is also worth mentioning that, in addition to exogenous auxin and cytokinin stimulus, wounding is an important trigger to enhance cell reprogramming in the two-step culture system, since root tissue without wounding fails to regenerate shoots even after the CIM-SIM treatment. Consistently, ectopic expression of *WIND1* complements shoot regeneration even from unwound tissue in the two-step tissue culture (Iwase et al. 2015).

## Cell Reprogramming by Protoplast Culture

Callus formation and whole plant regeneration from a single protoplast cell derived from a mature mesophyll cell are also used to study the reprogramming in Arabidopsis (Chupeau et al. 2013, Xu et al. 2021, Sakamoto et al. 2022). Compared to pericycle or pericycle-like cells, which retain a capacity for cell division for lateral root formation, mature mesophyll cells have several traits of more differentiated cells. For example, they do not divide in intact plants during normal development, and they have developed organelles such as chloroplasts and expanded vacuoles. Therefore, callus formation from a single mesophyll protoplast is a suitable model to study reprogramming from differentiated cells, where cell division reactivation and dramatic changes in organelle structures are indispensable.

Transcriptome analyses during protoplast cultures identified several key factors for regeneration, providing key insights into underlying molecular mechanisms. For example, the expression of *ALF4* is upregulated in the first 24 h after protoplast culture. The *alf4* loss-of-function mutants fail in protoplast division, demonstrating that *ALF4* is also essential for the reprogramming of protoplasts (Chupeau et al. 2013). Similar to this, the *arf7 afr19* double mutant shows clear defection of callus formation from protoplast (Sakamoto et al. 2022), suggesting that the auxin-induced lateral root formation/callus formation pathway is involved in the protoplast regeneration. *WUS* and *ESR1* transcription factors, also induced during protoplast culture, were required for regeneration as their mutants failed in callus formation, while their overexpression promoted regeneration (Xu et al. 2021).

Like wound-induced or auxin-induced callus formation, epigenetic regulation of reprogramming regulators plays a profound role in protoplast reprogramming. A previous microscopic analysis revealed that the heterochromatin underwent decondensation in the early phase of protoplast culture (Tessadori et al. 2007). Recently, Assay for Transposase-Accessible Chromatin sequencing (ATAC-seq) showed that global chromatin accessibility increases upon protoplast isolation and culture (Xu et al. 2021). Their study also showed that Trichostatin A treatment, an inhibitor for histone deacetylation, enhances protoplast regeneration (Xu et al. 2021). Consistently, treatment with Butyrolactone 3 (also called MB-3), a histone acetylation inhibitor, strongly suppresses cellular division activation in protoplasts (Sakamoto et al. 2022), suggesting that a global increase in chromatin accessibility by histone acetylation is a critical step for cell reprogramming from the differentiated plant cell. The transcriptomic analyses comparing MB-3-treated and non-treated protoplasts combined with chromatin immunoprecipitation (ChIP) analyses for histone modification identified key regulators of cell division reactivation during reprogramming and the mechanism of its transcriptional regulation. Auxin response triggered by auxin biosynthesis induces transcriptional activators of cell cycle G2/M phase–related genes, *MYB3R1* and *MYB3R4* (Haga et al. 2007), to re-enter the cell division phase during protoplast culture. This auxin biosynthesis is enhanced by the transcriptional activation of an enzyme for auxin biosynthesis, *YUCCA1* (*YUC1*), by the PLT3 and PLT5 transcription factors, which are regulated through histone acetylation upon protoplast culture (Sakamoto et al. 2022).

## Reprogramming of Differentiated Cells in Various Plant Species

Although the Arabidopsis research has made remarkable progress in cell reprogramming mechanisms, our understanding of how some differentiated plant cells maintain differentiated status and cell developmental plasticity simultaneously is still very limited. It will be necessary for the future to perform molecular analyses in experimental systems where reprogramming is observed from differentiated cells, as tissue culture approaches exemplified in **Table 2**.

Cell reprogramming from protoplast has been reported in plant tissue other than Arabidopsis mesophyll. Protoplast isolated from the epidermis of tobacco, which is morphologically distinct from mesophyll protoplasts, can also form calli that regenerate plants (Davey et al. 1974). Callus derived from root hair protoplasts of *Lotus corniculatus* can regenerate shoots and roots (Rasheed et al. 1990). Molecular studies on dynamic changes in the chromatin structure during protoplast isolation and culture are also performed with mesophyll protoplasts of tobacco (*Nicotiana tabacum*) (Williams et al. 2003, Avivi et al. 2004).

**Fig. 1:**
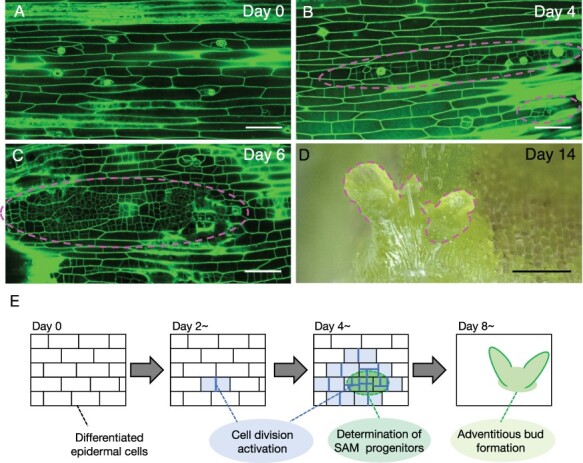
Reprogramming of epidermal cells in *T. fournieri.* (A–C) Activation of cell division in the Torenia stem culture system. Images show propidium iodide–stained epidermis before culture (A), after 4 d of culture (B) and after 6 d of culture (C). Dashed lines indicate foci with active cell division. (D) A stem explant cultured with cytokinin for 2 weeks. Dashed lines indicate adventitious buds. (E) A schematic illustration describing the process of shoot regeneration in the Torenia stem culture system. Scale bars = 100 µm (A–C), 500 µm (D). Photographs are modified from Morinaka et al. (2021).

In addition, differentiated cells of some species can also undergo reprogramming, leading to organ regeneration by tissue culture of explants under the treatment of phytohormones. These tissue culture systems are more technically straightforward than protoplast isolation and, thus, would be suitable models for understanding the reacquisition of pluripotency in differentiated cells and its proper regulation. For example, when a stem segment of *Torenia fournieri* is cultured on a medium (**Fig. 1**), direct shoot regeneration from the epidermis is induced efficiently (Chlyah 1974). Direct shoot regeneration from the epidermis was also reported in other species, such as *Begonia rex* and *Nautilocalyx lynchii* (Chlyah and Van 1975, Venverloo et al. 1983). It is also known that somatic embryogenesis can be induced by tissue culture in several species. When a leaf segment of orchardgrass (*Dactylis glomerata*) is cultured with auxin, somatic embryogenesis occurs directly from mesophyll cells (Conger et al. 1983). Somatic embryogenesis from mesophyll also occurs in leaf segments of *Cichorium* cultured with auxin and cytokinin (Dubois et al. 1991). Similar observations, e.g. somatic embryogenesis from hypocotyl epidermis in carrots (*Daucus carota*) and leaf epidermis of moth orchids (*Phalaenopsis amabilis*), are also reported (Nishiwaki et al. 2000, Kuo et al. 2005, Chen and Chang 2006).

## Uncovering of Cell Reprogramming Mechanisms by Historical Tissue Culture Systems

Transcriptomic analyses to uncover the global changes in gene expression profiles during the cell reprogramming from specific differentiated cells will be the first step to understanding molecular details of the reacquisition of pluripotency. With the Torenia stem culture system described earlier, we recently performed cytological and transcriptomic analyses by RNA-sequencing (RNA-seq) during cell reprogramming from epidermal cells to shoot stem cells (Morinaka et al. 2021). Physiological studies on this Torenia culture system in the 1970s–1990s investigated the effect of various growth regulators and found that cytokinin strongly promotes shoot regeneration (Kamada and Harada 1979, Tanimoto and Harada 1982, 1984). Consistently, our cytological analyses showed that cell division is activated after 2 d of culture in a cytokinin-dependent manner, and subsequently, shoot apical meristem (SAM) progenitors are established, leading to adventitious bud formation (**Fig. 1**). We also confirmed nucleus and nucleoli enlargement of the Torenia epidermal cell quantitatively at 2 d after the culture before the cell division started, especially in the cytokinin-treated condition (Morinaka et al. 2021). Chlyah pointed out that nuclei and nucleoli of the Torenia epidermal cells increased their volume before the commencement of cell division (Chlyah 1974). Similar nuclear and nucleolar enlargement was reported in other dedifferentiating plant cells (Feldman and Torrey 1977, Williams and Jordan 1980, Paul et al. 1989, Williams et al. 2003). This may bear more resemblance to totipotent embryonic callus cells than meristem stem cells, which tend to have smaller nucleoli with dense heterochromatin (Verdeil et al. 2007). In animals, embryonic stem cell pluripotency is also positively associated with a large hyperactive nucleolus, which is thought to play an important role in altering global genome architecture (Gupta and Santoro 2020). Ribosomal RNA biogenesis–dependent phase separation in the nucleoli of mouse stem cells can regulate cell fate via a key reprogramming regulator (Yu et al. 2021). Taken together, the nucleolar enlargement in the Torenia epidermis might be related to cell reprogramming accompanying chromosomal changes.

Using transcriptome analysis in combination with cytological analyses, we found three distinct phases with unique transcriptomic profiles during shoot regeneration from Torenia stem epidermal cells. The first phase includes rapid loss of the original stem features and acquisition of multiple organs/tissue signatures characterized by simultaneous gene expression normally expressed preferentially in the shoot apex, leaf or root. Interestingly, genes that are commonly upregulated in the three types of cell reprogramming, wound-induced callus formation, CIM-induced callus formation and protoplast-derived callus formation, are upregulated in this initial phase. This is followed by the second phase where the cytokinin-dependent transcriptional activation of genes related to cellular proliferation was observed. In the third phase, SAM regulatory genes were induced, leading to SAM formation (Morinaka et al. 2021).

On further careful examination of the transcriptomic data, we observed that the Torenia homologous genes for wound-induced reprogramming regulators, such as *WIND1, ESR1* and *ERF115*, are also induced immediately and constantly upon culture. The critical factor for the CIM-SIM and protoplast reprogramming, *ALF4*, is transiently expressed within 3 h after incubation on tissue culture media. On the other hand, the expression of homologs of root meristem regulators such as *WOX5, PLT1, PLT2* and *LBD16* was not induced during regeneration, implying that the wound-induced cell reprogramming pathway would play a major role in the cell reprogramming initiation of the Torenia epidermal cell rather than the callus formation via the lateral root formation pathway (Morinaka et al. 2021). Takeuchi et al. demonstrated that extra wounding on stem explants before culture increases regeneration in this system, supporting the idea that wounding stress has a promotive effect on triggering epidermal reprogramming (Takeuchi et al. 1985).

## Future Perspectives

**Fig. 2:**
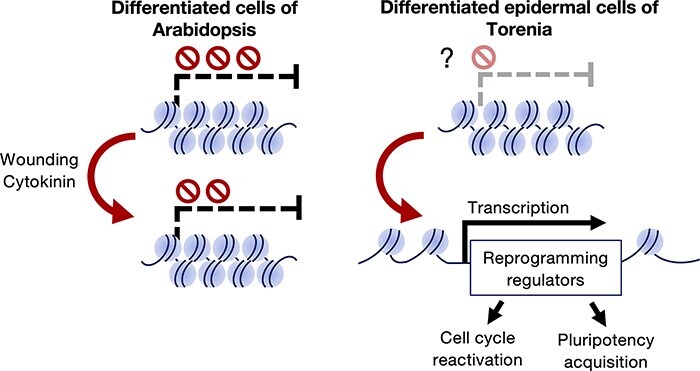
Hypothetical epigenetic regulation of reprogramming in differentiated cells. The induction of reprogramming regulators involved in cell cycle reactivation and stem cell formation is a key step of reprogramming. In differentiated cells of Arabidopsis, the expression of these regulators is tightly repressed by epigenetic regulation. On the other hand, in Torenia epidermal cells that maintain developmental plasticity after differentiation, the expression of reprogramming regulators is induced upon external stimuli such as wounding and cytokinin. Accumulating evidence from Arabidopsis and Torenia research implies that this induction is achieved by flexible epigenetic repression of these regulators that enables chromatin decondensation upon external stimuli.

In differentiated Arabidopsis cells, the expression of genes responsible for reprogramming is tightly repressed by epigenetic mechanisms (Ikeuchi et al. 2015). In contrast, the expression of such reprogramming genes might be suppressed more flexibly in differentiated cells that are more easily reprogrammable, such as Torenia epidermal cells (**Fig. 2**). It would be important to uncover the epigenetic regulatory mechanisms of the gene clusters that control stem cell conversion of differentiated cells in order to elucidate the molecular mechanisms underlying the ease of reprogramming of differentiated plant cells. The epidermis of the Torenia stem explants during the regeneration process is composed of heterogeneous cell populations, including cells that are reprogrammed into stem cells, cells that do not divide, cells that develop into SAM and cells that do not form SAMs (**Fig. 1**). Focusing on this cell heterogeneity and capturing differences in gene expression among various cell types will lead to the identification of gene networks that are critical for the transformation from differentiated cells into stem cells as well as the epigenetic alteration during formation of *de novo* SAM. Thanks to the progression of molecular biology and DNA sequencing technologies, we now have unprecedented access to genomic information of various ‘non-model’ plants (Unamba et al. 2015). This has allowed us to elucidate novel gene regulatory networks by RNA-seq from the non-model plants in tissue culture conditions such as the Torenia culture. We now have the opportunity to utilize advanced technologies, like histone modification by ChIP-seq and chromatin openness by the ATAC-seq at the single-cell level. These approaches will clarify the basis for the high regenerative capacity of plants comprehensively. It is also worth noting that studying stem cell reformation from differentiated cells has crucial implications in other biology since it can address how cells induce or maintain cell differentiation status and how cells exert pluripotency or totipotency. This knowledge will help us establish more efficient tools for organ regeneration.

## Data Availability

No new datasets were generated or analyzed in this study.
